# Appendicite aigue et grossesse extra utérine simultanée: a propos d'un cas rare

**DOI:** 10.11604/pamj.2013.14.35.2382

**Published:** 2013-01-24

**Authors:** Fatima Zohra Fdili Alaoui, Kamilia Laabadi, Hekmat Chaara, Hekmat Bouguern, Abdilah Melhouf

**Affiliations:** 1 Service de Gynécologie-Obstétrique II, CHU Hassan II, Université Mohamed Ben Abdelah, Fès, Maroc

**Keywords:** Appendicite aigue, grossesse extrautérine, hormone gonadotrophique chorionique, laparotomie, beta-HCG, acute appendicitis, ectopic pregnancy, chorionic gonadotropin hormone, laparotomy, beta-HCG

## Abstract

L'association d'une appendicite aigue et une grossesse extra utérine est rare, seulement une vingtaine de cas ont été rapportés dans la littérature. Nous présentons un cas d'une patiente de 27 ans opérée pour suspicion de grossesse extra utérine droite, le diagnostic d'appendicite aigue a été fait en peropératoire devant la constatation d'un appendice enflé accolé à la masse latéroutérine. A travers ce cas rapporté et une revue de la littérature, nous mettons le point sur la nécessité de penser à l'appendicite aigue associée devant un tableau évoquant une grossesse extra utérine surtout à droite en raison de la relation étiopathogénique entre ces deux pathologies responsables de douleurs aigues bien que leur association reste rare.

## Introduction

L'association d'une appendicite aigue (AA) à une grossesse ectopique est un fait rare; Depuis 1960, seulement 23 cas d'association d'AA et grossesse extra utérine ont été décrits et trois cas d'appendicite associée à une grossesse hétérotopique [1]. A travers ce cas d'association de grossesse extra utérine à une appendicite aigue chez une patiente de 27 ans, et une revue de la littérature, nous mettons le point sur les aspects étiopathogéniques, cliniques et paracliniques de cette association rare.

## Patient et observation

Il s'agit de Mme HK, âgée de 27 ans, mariée depuis 1 an et demi, nullipare admise dans notre formation pour prise en charge d'une douleur de la fosse iliaque droite apparue depuis 1 semaine associée à des métrorragies sur aménorrhée de 2 mois.

L'examen à l'admission trouve une patiente apyrétique, les conjonctives normocolorées, avec une tension artérielle à 10/6 et une fréquence cardiaque à 80 battements par minute. L'examen abdominal trouve une sensibilité pelvienne bilatérale accentuée à droite. L'examen gynécologique objective un col d'aspect normal, un saignement minime et noirâtre provenant de l'endocol, un utérus de taille normal avec un empâtement latéroutérin droit sensible sans cri de douglas.

**Figure 1:**
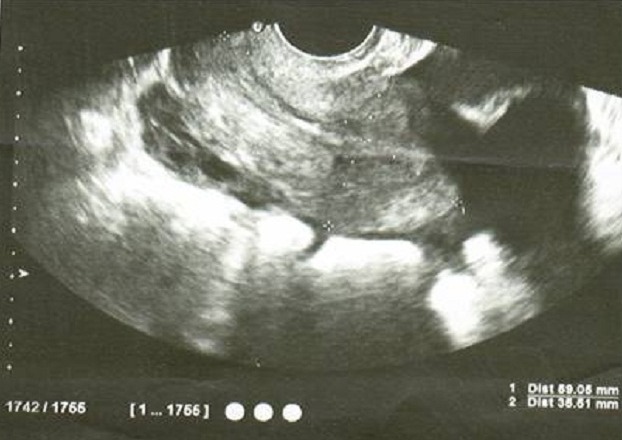
Échographie endovaginale de Mme H K montrant en coupe longitudinale un utérus de taille normale avec une ligne d'interface suivie jusqu'au fond

**Figure 2:**
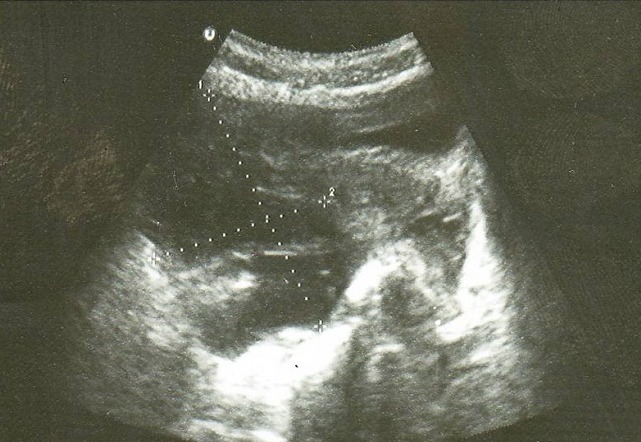
Echographie endovaginale de Mme HK montrant en coupe transversale une image latero utérine droite mal systématisée (89/60mm)

**Figure 3:**
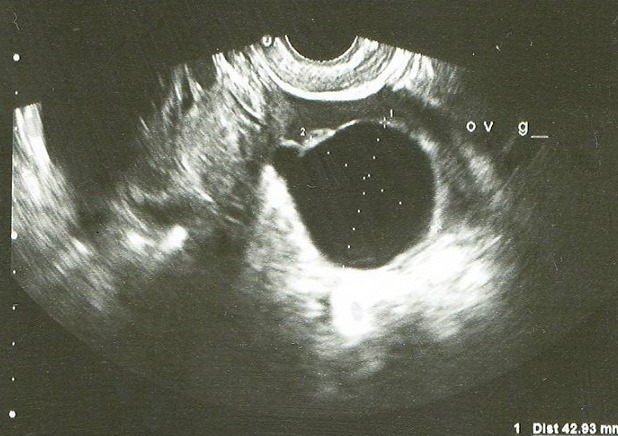
Echographie endovaginale de Mme HK montrant une image kystique simple de 42/42mm au dépend de l'ovaire gauche

A l'échographie pelvienne: l'utérus est de taille normal avec une ligne d'interface qui est suivie jusqu'au fond (Figure 1), présence d'une masse latéroutérine droite mal systématisée faisant 89/60mm (Figure 2), l'ovaire gauche est visualisé siège d'une image anéchogène kystique simple de 42/42mm (Figure 3). Para ailleurs un épanchement de moyenne abondance est noté. Le taux de β-HCG (hormone gonadotrophique chorionique) plasmatique est revenu positif à 1437 UI/l.

Le tableau évoque en premier le diagnostic de grossesse extra utérine droite. Le reste du bilan biologique révèle une hémoglobine à 11,4 g/dl, des globules blancs à 7530/mm^3^. La patiente bénéficie donc d'une minilaparotomie transversale: A l'exploration, présence d'un hémopéritoine de 700cc, avec découverte d'une grossesse extrautérine en voie d'organisation avec trompe droite boudinée, et perforée, d'où la décision de rélaiser une salpingectomie droite rétrograde. Le reste de l'exploration révèle des adhérences interhépatodia phragmatiques avec un appendice enflé accolé à la masse latéroutérine, une appendicectomie est réalisée. L'étude anatomopathologiaque révèle une grossesse tubaire perforée avec une appendicite aigue suppurée avec réaction péritonéale locale. Les suites post opératoires sont sans particularités et la patiente est déclarée sortante au 3^ème^ jour.

## Discussion

L'association d'appendicite aigue (AA) à une grossesse extrautérine (GEU) est rare. Depuis 1960, seulement 23 cas d'association d'AA et grossesse extra utérine ont été décrits et trois cas d'appendicite associée à une grossesse hétérotopique [1]. Les douleurs pelviennes aigues survenant dans un contexte gravidique peuvent avoir plusieurs étiologies à cause des modifications anatomiques au cours de la grossesse, les causes les plus fréquentes étant la torsion ovarienne, le fibrome en dégénérescence, le corps jaune hémorragique, rompu, l'abcès tubo ovarien, l'infection génitale. L'appendicite au cours de la grossesse survient avec une fréquence de 1/1500 grossesses; la grossesse ectopique quant à elle survient avec une fréquence de 16/1000 patientes [2].

Le risque de GEU augmente avec les antécédents de GEU, la chirurgie tubaire ou digestive, la stérilisation tubaire, la maladie inflammatoire pelvienne, le tabac, la prise de microprogestatifs, le port de dispositif intrautérin, le traitement de l'infertilité par les inducteurs de l'ovulation,la fécondation in vitro, le transfert de gamètes [3]. Jusqu'à présent, il n'existe pas de théorie unique sur l'étiologie de l'appendicite mais la combinaison entre l'obstruction luminale et /ou l'infection est retenue comme principal facteur éthiogénique.

La relation étiopathogénique entre l'AA et GEU responsables de douleurs aigues chez la femme est expliquée par l'inflammation péri appendiculaire causée par la GEU et responsable d'une colonisation bactérienne qui entrainerait l'appendicite aigue[4]. Dans le cas contraire, un antécédent d'AA qui a guéri spontanément, peut entrainer des lésions tubaires par des remaniements inflammatoires favorisant le développement d'une GEU [5]; 75% des GEU se situent ainsi à droite [2].

Au cours du premier trimestre, l'appendice est en position normale et la symptomatologie est celle d'une forme classique, la difficulté est de rapporter les vomissements soit à la grossesse soit à l'appendicite de même la température est rarement très élevée d'emblée.

Concernant l'examen clinique: la douleur provoquée siégeant au niveau de la fosse iliaque droite au point classique de Mac Burney, la douleur provoquée à la décompression brusque à ce niveau (signe de Blumberg), la douleur controlatérale provoquée au niveau de la fosse iliaque gauche (signe de Rovsing), la défense, la contracture pariétale; Tous ces signes orientant vers une appendicite aigue sont masqués par les signes de GEU beaucoup plus manifestes [6]. Le nombre de globules blancs et la vitesse de sédimentation peuvent être physiologiquement élevés au cours de la grossesse en l'absence d'infection.

Les progrès concernant l'échographie abdomino pelvienne surtout transvaginale devenue à haute fréquence associée au dosage du taux de ß HCG plasmatique quantitatif ont facilité le diagnostic d'une grossesse extrautérine avant même l'apparition des signes cliniques. Par ailleurs le manque de signes échographiques spécifiques en faveur d'une appendicite aigue doit toujours inciter le praticien à évoquer et rechercher une association possible de GEU et AA devant une douleur pelvienne aigue chez une patiente enceinte [7]. Notre patiente avait des signes typiques en faveur de GEU mais non spécifiques d'AA qui n'a été diagnostiquée qu'en peropératoire.

La confirmation du diagnostic et la prise en charge de la GEU associée à une AA peut se faire en effectuant une laparoscopie, minilaparoscopie avant d'avoir recours à une laparotomie comme de nombreux auteurs le recommandent [8].

## Conclusion

Chaque praticien doit être vigilant quant à l'association rare mais possible d'une appendicite aigue à une grossesse extra utérine devant une douleur aigue dans un contexte gravidique. La relation inter étiologique entre ces deux entités est expliquée par la progression du processus inflammatoire et infectieux par contigüité.

Par conséquent, il est important en l'absence de signes cliniques spécifiques en préopératoire, de vérifier l'appendice au cours d'une intervention pour grossesse extra utérine surtout droite afin d'éliminer une appendicite concomitante.
